# Effects of elastic taping on kyphosis and body balance in the elderly: a randomized crossover study

**DOI:** 10.1038/s41598-024-52047-x

**Published:** 2024-01-16

**Authors:** Surapa Tangpakkakul, Nuttaset Manimmanakorn, Apiwan Manimmanakorn, Ratana Vichiansiri, Michael J. Hamlin

**Affiliations:** 1 Department of Rehabilitation Medicine, Faculty of Medicine, Khon Kaen University, Khon Kaen, Thailand; 2 Department of Physiology, Faculty of Medicine, Khon Kaen University, Khon Kaen, Thailand; 3 Department of Tourism, Sport and Society, Lincoln University, Christchurch, New Zealand

**Keywords:** Medical research, Health care, Geriatrics

## Abstract

Kyphosis produces abnormal posture and reduced body balance in the elderly. Elastic tape may be useful at improving kyphotic posture and body balance. This study aims to evaluate the effects of elastic taping on kyphosis and body balance in the elderly. Ten elderly participants with degenerative kyphotic posture were recruited and randomly assigned to two groups (back taped with stretched elastic tape for 15 min and back taped with non-stretched elastic tape for 15 min). After a 1-h washout period, the groups were swapped over to receive the other intervention. The outcomes measured after each taping technique were Cobb’s angle measurement by inclinometer, perceived pain, and balance measurements by single leg stance test, time up and go test, center of gravity alignment (COG) and modified clinical test of sensory interaction on balance test (mCTSIB). There was a significant reduction in kyphotic angle and back pain in both the stretched and non-stretched taping groups (*p* < 0.05). We also found both taping techniques significantly reduced sway velocity on a foam surface with eyes closed and open (*p* < 0.05). However, there was no significant difference between taping groups for kyphotic angle, pain reduction or balance. The application of 15 min of stretched and non-stretched elastic tape in the elderly reduced kyphotic angle, back pain, and sway velocity while standing on foam surface in the mCTSIB test. If these changes persist over the long term (days and weeks) taping may be a useful intervention for elderly patients with kyphosis.

## Introduction

Kyphosis is a common health problem in the elderly with approximately 20–40% of the population over 60 years of age suffering from this condition^1^. A kyphotic posture can result from several factors including muscle weakness, spinal degeneration, and osteoporotic change^2^. The kyphotic or stooped posture tends to bend the upper body forward, thereby relocating the individuals center of gravity, escalating balance problems, which can increase the risk of falls^3,4^. Traditional ways to reduce kyphotic posture and thereby improve balance, reduce risk of falls and improve the quality of life are specific back exercises, postural retraining, and spinal orthosis^5–7^.

Therapeutic tape has also been found to be an effective method in kyphotic angle reduction. For example, Greig et al.^8^ conducted a randomized control trial in 15 women with thoracic kyphosis from spinal osteoporotic fractures and compared two levels of therapeutic rigid taping (no-tension taping versus tension taping) with a control group (no taping). Results indicated that the tension-taped group had a significantly decreased kyphotic angle compared to no-tension tape and no tape groups. There was no taping effect on trunk muscle electromyography or force plate-derived balance parameters suggesting that the kyphotic angle improvement was due to structural re-alignment and mechanical support^8^.

While the early study of Greig et al.^8^ used non-elastic tape, more recently elastic tape (which can be stretched up to 140–150% of its length) has also been used in therapeutic environments. Elastic tape has been commonly used for relieving pain by stimulating the somatosensory system, improving tissue alignment, and activating muscle performance^9–13^. Elastic tape, when attached to the skin causes skin convolution which lifts the skin and fascia up, thereby creating gaps under the skin, which increases interstitial space, stimulating blood and lymph flow^13^.

It is also thought that elastic tape increases proprioceptive feedback from tissue underneath the skin which may influence subsequent central nervous system activity^14–16^. Using such tape applied diagonally from the acromioclavicular joint to T6 after posture correction (Greig et al.^8^) is likely to add stretch to the upper trapezius, back extensor, and scapular retractor muscles which may stimulate receptors in these muscles and other mechanoreceptors involved in detection of stretch, load, pressure, and shear force^16,17^. This increased innervation from the many receptors results in altered gamma motor neuron firing from the central nervous system and a change in muscle tone, thereby altering muscle function and perhaps muscle strength^16,17^. If the upregulation of the proprioceptive information to the central nervous system reaches consciousness, there may also be a change in the participants awareness of this information and a concerted effort by the participant to straighten their back which would shift their center of gravity backwards and thereby increase their function and balance ability^18,19^.

A previous study has found that applying stretched elastic tape on the back of cerebral palsy patients for 4 weeks reduced kyphotic curvature compared to a control group (no taping)^20^. However, the effect of elastic tape for kyphosis reduction in the elderly has not been investigated. Reducing kyphotic problems may result in less pain and improve quality of life for the elderly^21^.

The tension of elastic tape is exponentially correlated to force generation^22^. However, the effects of different tension degrees of taping to neuromuscular stimulation are still equivocal. Shakeri et al.^23^ found elastic tape increased muscle strength, but the different tension was not related to increased muscle strength. Chen et al.^22^ showed the different tension had no effect on modulating sensorimotor activity. Craighead et al.^24^ showed the microvascular skin blood flow increased after applied Kinesio tape, but it was not related to degree of tape tension. The tension degree of elastic tape in correcting kyphotic angle have not been evaluated.

Therefore, we still do not know what the effects of stretched elastic taping in reducing kyphosis and subsequently improving body balance in the elderly compared to non-stretched taping would be. If stretched elastic taping has more beneficial effect compared to non-stretched taping, it may be used as an alternative treatment for kyphosis in the elderly.

This study aims to compare the acute effects of elastic tape with and without stretch on reducing kyphotic angle and improving body balance in elderly participants.

## Methods

This crossover designed study was conducted at Srinagarind Hospital, Khon Kaen University, Thailand (from 3 December 2018 to 30 March 2019). The study was approved by the Khon Kaen University Human Ethical Committee (IRB No. HE611340) and all methods were performed in accordance with the relevant guidelines and regulations of the Declaration of Helsinki. The study was registered in the Thai Clinical Trial Registry (www.thaiclinicaltrials.org, TCTR20181211003, 11/12/2018). Informed consent was obtained from all participants and/or their legal guardians.

### Participants

Participants had to meet the following criteria: (1) aged 60 years and over, (2) suffered from postural thoracic kyphosis of at least 20 degrees, (3) able to walk with or without gait aids more than 6 m, (4) have a back pain score less than 5 on an analogue–digital scale from 1 to 10. Participants were excluded from the study if they suffered from the following, (1) non-postural kyphosis such as malignancy, tuberculosis spondylitis or ankylosing spondylitis, (2) history of skin allergy with taping, (3) musculoskeletal pain such as osteoarthritis of the hip, knee or ankle.

All participants who were invited into this study by authors ST and NM were randomly allocated to the alternative taping techniques by computer program using a simple randomization method. The sequence allocation was concealed by sealed and opaque envelopes. The randomization and allocation were performed by a research assistant who was blinded to the group information.

**Figure 1:**
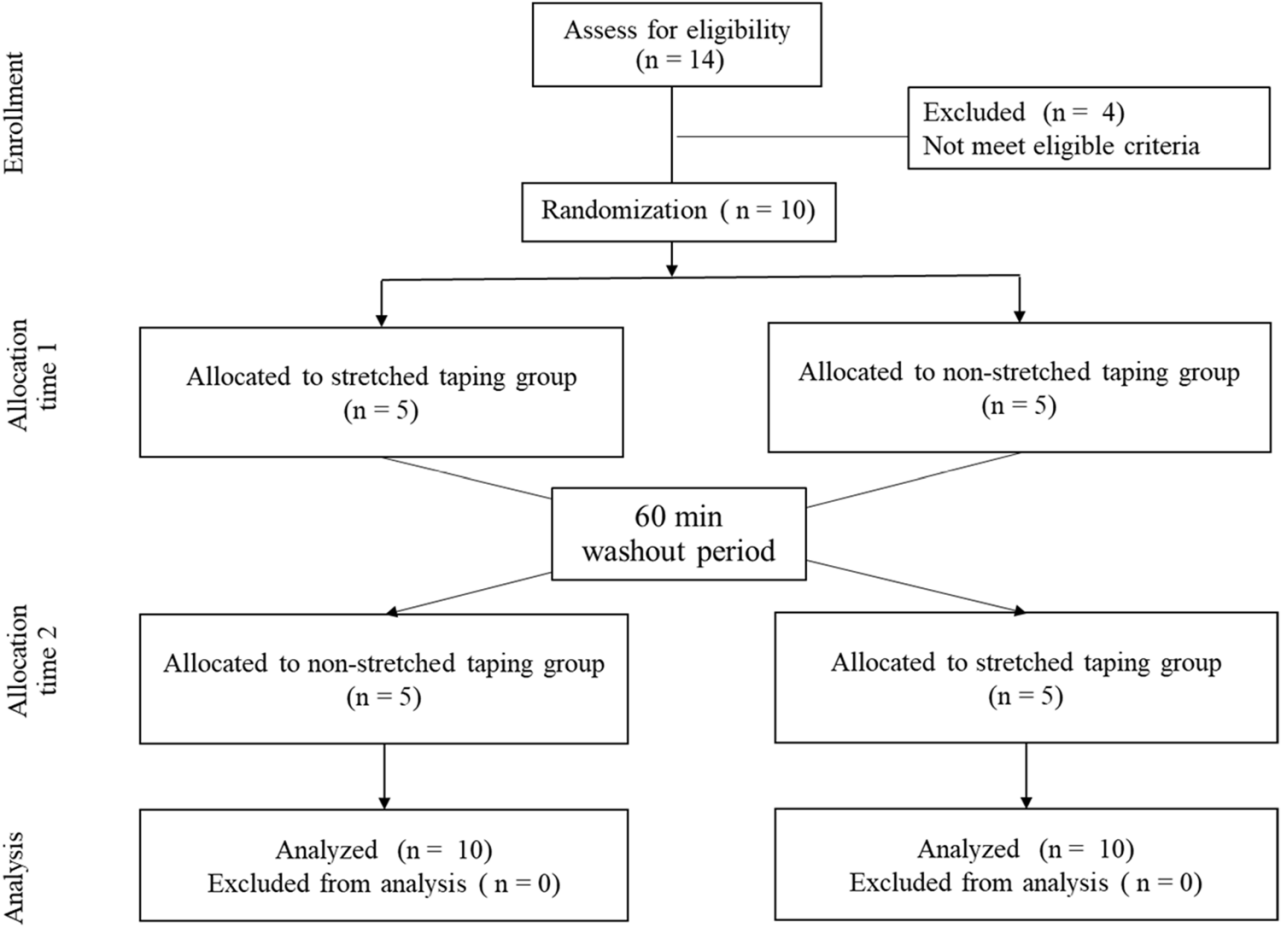
Consort diagram of this study.

**Figure 2:**
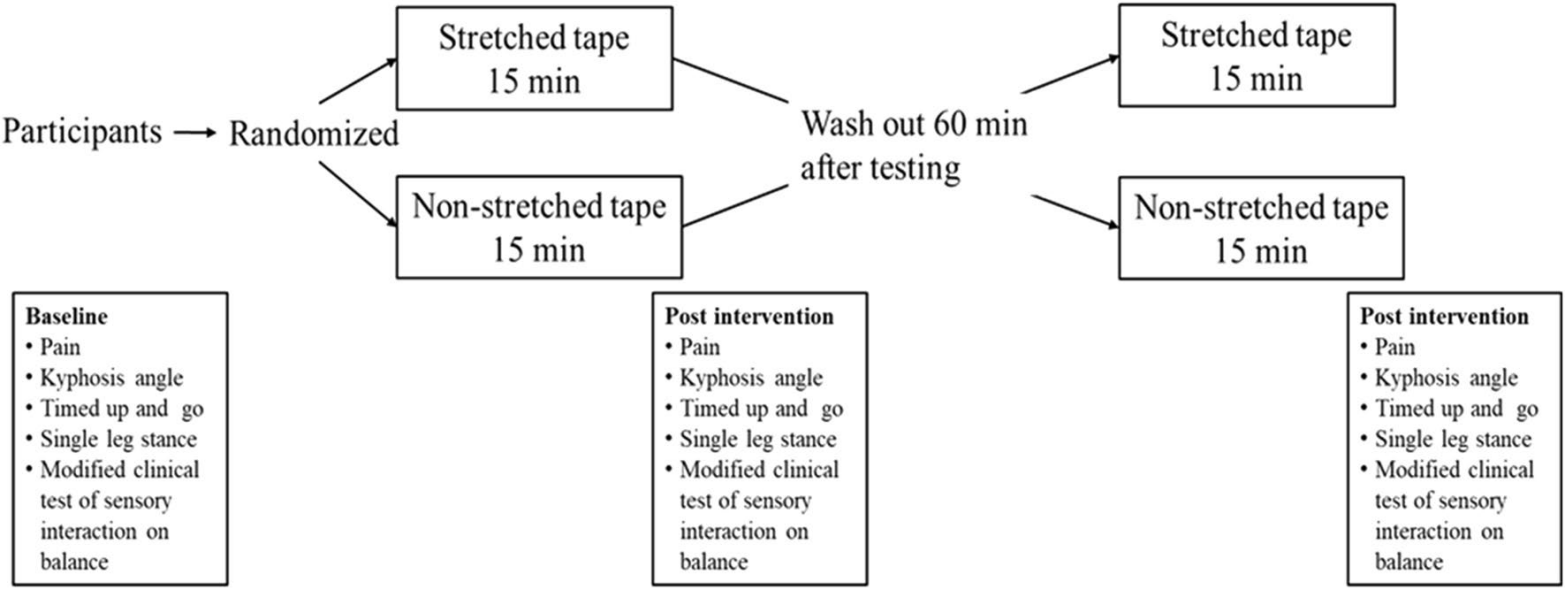
Diagram of this study.

Ten participants, 2 male and 8 female, aged 76.1 ± 6.8 years (mean ± SD) were included in this study and randomized into 2 groups: group 1, stretched elastic tape and group 2, non-stretched elastic tape. All participants were interviewed and evaluated during a pre-intervention test. The participants received either stretched elastic tape or non-stretched elastic tape for 15 min and then the post-intervention test was performed. The participants remained blinded to the taping technique they received. After completing the post-intervention tests which took approximately 30 min, participant groups were swapped and given a wash-out period of 60 min where they sat and relaxed before starting the other intervention. After 15 min taping, the participants completed the post-intervention tests again in their new group. Therefore, after the crossover, data from 20 participants was available (n = 10 for stretched tape and n = 10 for non-stretched tape). No participants dropped out of the study (Figs. 1 and 2).

### Intervention

**Figure 3:**
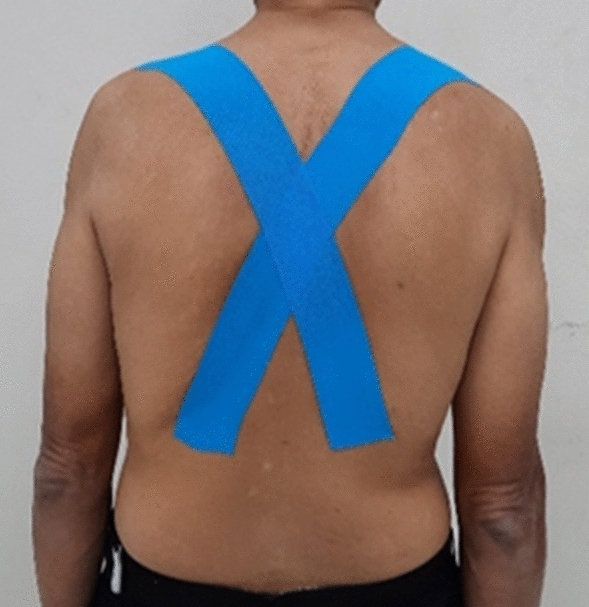
Taping technique on the upper back of participants in upright standing.

Elastic tape (Kinesio tape, Kinesio® Tex Classic, Kinesio Holding Corporation, New Mexico, USA) 5 cm in width, was applied on the upper back of participants by a certified physiotherapist who was not involved in the study. The technique of elastic taping was adapted from the protocol of Greig’s study^8^. When applying the tape, the participants were in an upright standing position with both arms along the side of the body in relaxed position. Taping was applied from a starting point at the anterior aspect of the acromioclavicular joint and was applied across the spinous process at T6 with the tape ending around T12 paraspinal muscle. In the stretched-tape group the elastic tape was applied with a stretch of approximately 150%, while in the un-stretched tape group, elastic tape was placed in the same fashion but was not stretched (Fig. 3).

### Outcome measurements

The outcomes of this study were kyphotic angle, body balance (single leg stance test), functional mobility (timed up and go test), modified clinical test of sensory interaction on balance (mCTSIB), and pain score. The participants were assessed by a blinded assessor before taping and 15 min after taping (while tape remained on the back) in standing position.

**Figure 4:**
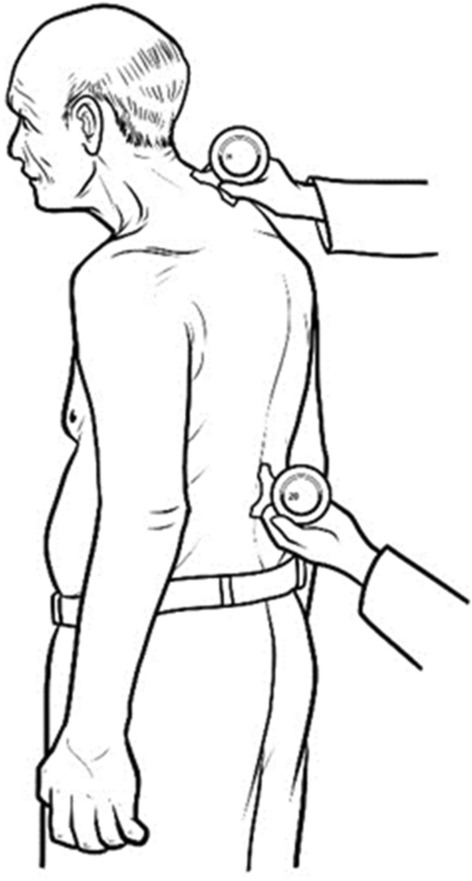
Measuring Cobb’s angle by using inclinometer.

The kyphotic angle was measured by the Cobb’s method using a gravity-dependent (analogue) inclinometer (Baseline Bubble® Inclinometer, Fabrication Enterprises Inc., NY, USA). During kyphotic angle measurement the participants were in a resting upright standing position on a flat surface, both legs aligned, and both arms relaxed alongside the body. The researcher put a cloth over the participants body apart from the area to be measured to blind the assessor. The evaluator palpitated the most prominent spinous process at C7 where the front foot of the inclinometer was positioned, and the first inclinometer angle (A1) was recorded. Similarly, the prominent spinous process of T12 was identified and the rear foot of the inclinometer was positioned, and the second inclinometer angle (A2) was recorded (Fig. 4). The summation of two angles (A1 + A2) was the kyphotic angle to be analyzed. The measurement was performed two times and the average was recorded. Hunter et al.^25^ showed the kyphosis angle measured from the inclinometer had a strong association to the modified Cobb angle measured from film X-ray. The manual inclinometer is a reliable instrument for thoracic kyphosis measurement that had excellent intrarater (ICC = 0.92) and interrater (ICC = 0.90) reliability^26^. Our measurement also found the excellent intrarater reliability (ICC = 0.96). The changing of the Cobb’s angle of at least 5° was determined as the minimal detectable difference^27^.

The degree of overall back pain was evaluated by a visual analog score (VAS). The participants performed self- assessment by rating back pain score from 0 for no pain to 10 for the worst pain. The visual analog score is a reliable and valid estimate of pain^28^.

Static body balance was evaluated by a single leg stance test that has excellent test–retest reliability and discriminant validity in the elderly subjects^29^. When the single leg stance test was performed, the participants were instructed to stand on their preferred leg as long as possible. The single leg stance test was timed when one foot was lifted from the floor until the same foot touched the ground or the other leg; no hand-held support was allowed during the test. The test was repeated 3 times, with a 5-min rest between each test, and the average time of the 3 trials was taken for analysis.

Functional mobility was assessed by the timed up and go test that also has excellent reliability and validity^29^. The participants sat in a chair with arms on the chair arm rests. The participants were instructed to rise from the chair, walk 3 m at a comfortable pace to a mark placed on the floor, turn 180 degrees at the 3 m mark, walk back to the starting point, and return to sitting in the chair. The time was recorded from starting to get up and ended when sitting back on the chair. The best of 3 trials was recorded after 5 min recovery was given between each trial. The average of 3 trials was analyzed.

Standing balance was objectively evaluated by measuring change in the center of gravity (COG) using force sensors underneath a platform of computerized dynamic posturography (NeuroCom Balance Manager®, a division of Natus, Oregon, USA). The modified clinical test of sensory interaction on balance (mCTSIB) was performed in this study to evaluate the mean COG sway velocity. According to Neurocom Balance Manager® measurement, sway velocity was defined as the mean speed of movement of the COG during the testing period^30^. The mean COG sway velocity is one of the most widely used parameters for balance testing^31,32^. We measured this in the standing position under four conditions: eyes open firm surface, eyes closed firm surface, eyes open foam surface, and eyes closed foam surface. The four conditions were performed to evaluate static balance when the visual or/and somatosensory input were eliminated. The participants took off their shoes and stood on a force plate for 30 s 3 times in each standing condition, and the average of mean COG sway velocity was recorded and analyzed.

The outcomes assessment started with rating overall back pain and measuring kyphotic angle, followed by single leg stance test, timed up and go test, and mCTSIB test with 5 min rest between each series of tests.

### Statistical analysis

The statistical analysis was performed by STATA version 10.1 (StataCorp LP, College Station, Tx, USA). The descriptive data is presented as mean ± SD. To compare outcomes between two groups (kyphotic angle, single leg stance, timed up and go, pain score, COG alignment, mCTSIB,), the statistical analysis for crossover design, a generalized estimating equation (GEE) was used with adjusted sequence effect, period effect and carry-over effect. The statistically significant difference was determined at p value less than 0.05.

The sample size was estimated based on crossover design study with beta error 0.2, alpha error 0.05 and a statistical significance P < 0.05. The mean difference of the outcome (kyphosis angle) from a previous study^8^ was 5.2 degrees, standard deviation was 4.9 degrees, and the superiority margin was 2 degrees^33^. Therefore, the calculated total participant numbers of each group were 10.

## Results

The characteristics of included participants are presented in Table 1. There was no significant difference in baseline data of all outcomes between stretched and non-stretched tape groups. The stretched elastic tape group significantly decreased kyphosis angle by 7.1 ± 3.2 degrees, mean ± SD, p-value < 0.001, while the non-stretched group also decreased kyphosis angle by 7.8 ± 7.1 degrees, p-value = 0.003 and there was no significant difference in kyphosis angle reduction between both groups (p = 0.944). Similarly, the pain score decreased significantly in both groups (by 1.2 ± 1.0, p = 0.023 for stretched elastic tape group and 1.1 ± 1.3, p = 0.020 for non-stretched group), once the tape was applied with no difference between the groups (p = 0.131).

**Table 1:** Demographic data of participants (n = 10).

Gender, no. (%)	Female	8 (80)
	Male	2 (20)
Age (year), mean (SD)		76.1 (6.8)
Weight (kg), mean (SD)		54.0 (10.0)
Height (cm), mean (SD)		150.8 (7.9)
BMI (kg m^−2^), mean (SD)		23.7 (4.0)
Underlying disease, no. (%)	DM	2 (20)
	HT	3 (30)
Walking ability	Independent	5 (50)
	Single cane	3 (30)
	Tripod cane	1 (10)
	Wheel walker	1 (10)
Number of falls in previous year, no. (%)	0	9 (90)
	1	1 (10)
History of using spinal orthosis, no. (%)		0 (0)
History of postural retraining, no. (%)		0 (0)
Cobb’s angle (^o^), mean (SD)		24.6 (9.3)

BMI, body mass index; DM, diabetic mellitus; HT, hypertension.

There was no significant change in the balance test (single leg stance test), functional mobility (timed up and go test) and COG alignment on X and Y axis from baseline to post-test in both groups and no significant difference between two groups (Table 2).

**Table 2:** Outcome variables before and after taping in the stretched and non-stretched groups.

Outcomes	Stretched taping (n = 10)			Non-stretched taping (n = 10)			Mean difference ± 95%CI	p-value between groups
	Pre-treatment mean (SD)	Post-treatment mean (SD)	p-value	Pre-treatment mean (SD)	Post-treatment mean (SD)	p-value		
Cobb’s angle (degree)	33.30 (19.8)	26.20 (20.5)	< 0.001*	34.8 (18.6)	24.90 (17.0)	0.003*	0.80 ± 22.2	0.944
Single-leg-stance (s)	15.30 (15.5)	15.83 (15.5)	0.606	16.40 (19.1)	22.60 (24.6)	0.213	− 1.67 ± 22.3	0.884
Time-up-and-go test (s)	21.40 (13.0)	22.60 (15.5)	0.373	21.30 (12.8)	20.70 (10.9)	0.515	8.40 ± 14.5	0.257
Pain score (VAS)	3.48 (1.1)	2.27 (1.4)	0.023*	3.62 (0.9)	2.49 (1.6)	0.020*	0.96 ± 14.5	0.131
Modified CTSIB Sway velocity (deg s^−1^)								
Firm-EO	0.58 (0.2)	0.63 (0.2)	0.276	0.59 (0.2)	0.63 (0.2)	0.539	0.002 ± 0.2	0.984
Firm-EC	0.70 (0.2)	0.71 (0.2)	0.739	0.67 (0.2)	0.71 (0.2)	0.264	− 0.09 ± 0.2	0.456
Foam-EO	1.11 (0.3)	1.00 (0.3)	0.016*	1.17 (0.3)	1.05 (0.3)	0.032*	− 0.32 ± 0.3	0.052
Foam-EC	1.80 (0.3)	1.67 (0.5)	0.028*	1.76 (0.3)	1.55 (0.4)	0.043*	− 0.52 ± 0.3	< 0.001^#^
Center of Gravity (COG) alignment (degree)								
Firm-EO: X axis	− 0.18 (0.8)	− 0.13 (0.9)	0.837	− 0.33 (− 0.4)	− 0.26 (1.2)	0.862	− 0.18 ± 0.9	0.701
Y axis	0.24 (0.7)	0.14 (1.1)	0.801	0.62 (1.5)	− 0.07 (0.8)	0.056	− 0.42 ± 1.2	0.485
Firm-EC: X axis	− 0.10 (0.8)	− 0.25 (0.9)	0.526	− 0.3 (0.7)	− 0.23 (1.0)	0.859	− 0.12 ± 0.8	0.772
Y axis	0.72 (1.2)	0.39 (1.0)	0.338	0.61 (2)	0.63 (1.4)	0.969	− 0.16 ± 1.7	0.854
Foam-EO: X axis	0.25 (0.6)	0.18 (0.7)	0.794	0.22 (1.3)	− 0.12 (1.0)	0.383	0.26 ± 1.1	0.636
Y axis	1.25 (0.9)	0.71 (0.8)	0.209	0.87 (0.9)	0.91 (1.1)	0.939	0.18 ± 1.0	0.737
Foam-EC: X axis	− 0.03 (0.7)	− 0.23 (0.5)	0.306	− 0.16 (1.1)	− 0.32 (0.9)	0.686	0.82 ± 0.9	0.086
Y axis	1.18 (1.1)	1.05 (0.9)	0.807	1.36 (1.4)	0.97 (1.0)	0.348	− 1.02 ± 1.2	0.098

Data are mean ± SD for pre and post-taping and mean ± 95% confidence interval for mean difference between two groups. VAS, Visual analog score; CTSIB, Clinical test of sensory interaction on balance; EO, eye open; EC, eye close.

*Significant difference compared within group (pre vs post), p < 0.05.

^#^Significant difference compared between two groups, p < 0.05.

For clinical test of sensory interaction in balance, the COG sway velocity significantly decreased in both groups compared to baseline while standing on a foam surface in the eyes open condition (p = 0.016 for stretched group and p = 0.032 for non-stretched group) and eyes closed condition (p = 0.028 for stretched group and p = 0.043 for non-stretched group). There was a significant reduction of the mean COG sway velocity while standing on a foam surface with eyes closed in the non-stretched elastic tape compared to stretched tape groups (p < 0.001), but there was no difference between two groups with eyes open (p = 0.052). There was no significant difference within groups and between groups for the mean COG sway velocity while standing on a firm surface with eyes open (p = 0.276 for stretched group and p = 0.539 for non-stretched group) or eyes closed (p = 0.739 for stretched group and p = 0.264 for non-stretched group). Finally, there was no reported skin allergy or skin irritation from wearing the tape by participants in the two groups.

There was no carry-over, sequence, and period effect (p > 0.05) on all outcome parameters except COG sway velocity standing on foam surface with eyes closed that had carry-over, sequence, and period effects (p < 0.001). However, the statistical analysis was adjusted to the carry-over, sequence, and period effect on this parameter.

## Discussion

This study showed that both elastic and non-elastic tape improved kyphotic angle and back pain acutely after 15 min of applying the tape with no statistically significant difference between the two methods. Taping for kyphosis did not change measures of body balance (single leg stance and timed up and go test) or COG alignment in the X and Y axis, but both taping regimes decreased the mean COG sway velocity during standing on the foam with eyes open and eyes closed.

Previous research (Greig et al.^8^) found that non-elastic therapeutic tape significantly reduced kyphotic angle by 5.3 ± 0.9% compared to control tape (no tape) 2.2 ± 0.8%. In addition, Prabhu et al.^34^ reported an improvement of kyphotic angle after Kinesio taping in an uncontrolled trial. Our study confirmed both stretched and non-stretched elastic taping reduced kyphotic angle by about 7 degrees. Mechanisms that may be involved in the improved kyphotic (Cobb’s) angle found in the participants of this study include increased mechanical support from the tape itself, or increased back extensor muscle activity (or both)^8^. Previous research has indicated elastic tape can increase joint stability, improve the direction of joint movement, and activate muscle contraction which may help to improve joint angle^13^. However, the current study found the stretched elastic tape was not superior to non-stretched elastic taping in the participants of this study. Putting stretch on the tape increases the elastic recoil of the tape which thereby generates more force on the taped structures. In this instance, more force did not result in better kyphotic angle improvement in the short-term. We suggest that perhaps the un-stretched tape also has some elastic effect which would probably decline over time. Future research should look at the differences in stretched and non-stretched tape over longer periods of time (as compared to the 15 min in this study). Alternatively, perhaps the attachment of tape (either stretched or non-stretched) to the participants back was enough to cause an increase in proprioceptive feedback which might have resulted in a conscious effort by the participant to correct the kyphosis. Unfortunately, we did not include a control group (no tape) which would have been useful in uncovering any such placebo effect of adding tape to the participants back. Future studies should include a control group to account for any placebo effects.

Previous research reported elastic tape reduced back pain compared to a control group with placebo taping^35,36^. Artioli et al.^36^ conducted a systematic review on the subject and concluded elastic tape possibly reduced back pain. More recently Li et al.^37^ conducted a meta-analysis and reported that elastic tape did not reduce chronic low back pain any more than placebo taping. Our study found the reduction in pain with taping (whether stretched or non-stretched) was similar and is probably due to improving joint angle and thereby reducing the cause of the pain. Others have also suggested taping can stimulate large afferent fibers^38^, increase neural feedback^12^, improve proprioception and increase muscle contraction to support surrounding tissue^39–42^, which may all help with reduction in pain stimuli or pain propagation. Further research is required to determine which of these mechanisms might be at work with the taping used in this study.

Previous research has suggested that therapeutic taping may stimulate muscle contraction and increase proprioception sensation^39–42^ which may improve body balance^43,44^. However, when Kinesio taping was applied on kyphotic patients in previous studies, the taping did not improve body balance^8,45^ and or COG alignment in the X and Y axis. The result of our study was similar to previous research indicating that Kinesio taping on kyphotic patients did not improve single leg stance performance or timed up and go time in the elderly. Important parameters of taping such as technique, location and duration may affect any influence taping has on body balance improvement and we recommend these parameters should be investigated in future studies.

This study found both stretched and non-stretched taping improved the modified clinical test of sensory interaction in balance (mCTSIB) or reduced the COG sway velocity during standing on foam surfaces with eyes open and eyes closed. While standing on the foam surface, the somatosensory input may be compromised^46,47^. Therefore, both taping techniques may have increased somatosensory stimuli in the body to reduce body sway, however this remains speculative until confirmed by further research. When standing with eyes closed (diminished visual perception), the non-stretched elastic taping group significantly reduced body sway velocity compared to the stretched tape group. Perhaps the increased force on body structures with stretched taping increased the focus of the participants on the tape rather than on maintaining their balance or body sway. Again, this needs to be evaluated in further studies with larger sample sizes.

### Limitations

A limitation of this study is the sample size which was relatively small, but given the fact that we had to have patients with existing kyphosis, only 10 subjects were available to us at the time of the study. The results of this study should be considered preliminary until substantiated with further research with larger sample sizes. In addition, this study was conducted on acute effects of both types of taping after being applied for 15 min, therefore, the results may not indicate what may happen after chronic or long-term taping (having the tape on for 1 or more days). Finally, there was no control group in this study (i.e. no tape group), therefore, the placebo effect of placing tape on the back should be considered.

## Conclusion

Both non-stretched and stretched elastic taping improved kyphotic angle and relieved back pain in elderly people and there was no difference in the effectiveness of taping between the groups. However, there was no improvement of body balance after taping to correct the kyphotic angle. Applying tape (either stretched or non-stretched) on a kyphotic back may improve kyphotic angle, reduce back pain and improve balance (reduce sway) in the short-term.

This research now needs to be replicated with larger subject numbers over a longer time to see if these improvements result in improved quality of life and reduced falls incidence in elderly people.

## Data Availability

The dataset and data analysis used in this study are available from the corresponding author on reasonable request.

## Abbreviations

mCTSIB: Modified clinical test of sensory interaction on balance test
COG: Center of gravity
SD: Standard deviation
GEE: Generalized estimating equation
